# Livelihood Vulnerability of Marine Fishermen to Multi-Stresses under the Vessel Buyback and Fishermen Transfer Programs in China: The Case of Zhoushan City, Zhejiang Province

**DOI:** 10.3390/ijerph17030765

**Published:** 2020-01-25

**Authors:** Qi Chen, Hongyan Su, Xuan Yu, Qiuguang Hu

**Affiliations:** 1 School of Business, Ningbo University, Ningbo 315211, China; 1511091887@nbu.edu.cn (X.Y.); huqiuguang@nbu.edu.cn (Q.H.); 2 School of Environment & Natural Resources, Renmin University of China, Beijing 100872, China

**Keywords:** vessel buyback and fishermen transfer programs, marine fishermen, livelihood vulnerability, livelihood assets

## Abstract

In the context of vessel buyback and fishermen transfer, some traditional marine fishermen changed their profession and turned to other related industries such as mariculture, fish processing, and recreational fishery. Studying the livelihood vulnerability of different types of fishermen is an important basis to help fishermen rebuild sustainable livelihoods. This paper developed a framework of a fishermen’s livelihood vulnerability assessment under multi-stresses, and then conducted an empirical analysis based on a survey in Zhoushan City, Zhejiang Province, China. Finally, the determinants of livelihood vulnerability were analyzed by a regression tree model. Results showed that fishermen with a high level of vulnerability accounted for about 37.35%, and they had some unique characteristics such as advanced age, low education levels. Although converted fishermen faced fewer exposure risks than non-converted fishermen, they eventually showed higher vulnerability due to poor adaptive ability. The livelihood vulnerability of fishermen engaged in recreational fisheries was relatively low, while that of fishermen engaged in non-fisheries was quite different from each other. The results of the regression tree analysis showed that the number of household income sources, whether they converted or not, impacts of disturbances, and whether they were equipped with fishery facilities could influence the fishermen’s livelihood vulnerability. The government should pay more attention to the fishermen whose family income source was single, and the converted fishermen whose productive physical capital was scare.

## 1. Introduction

The issue of the sustainable development of global marine fisheries has been discussed much since the late 1980s, which has given rise to some attention on the fishermen’s livelihood. Traditional marine fisheries were frequently characterized as “the occupation of last resort” and fishermen as “the poorest of poor” under the effect of multiple natural and anthropogenic threats [1].

In recent years, China’s marine fisheries have expanded rapidly and made a significant contribution to food security, creating employment, and sustaining the coastal economy in China [2]. However, overfishing, climate change, sea land reclamation activities, and marine pollution cause substantial reductions in local stocks of marine fish, which were considered as significant impacts negatively influencing marine fishermen’s livelihoods [3,4,5]. To reduce overcapacity in fisheries and conserve fishery resources, in 1987 the Bureau of Fisheries under the Ministry of Agriculture of China (MOA) (Beijing, China) proposed the ‘Double Control’ system, referring to the control of both the total number of marine engine-powered fishing vessels and their total engine power [6]. In order to lead traditional fishermen to change their careers and engage in other industries, the Ministry of Finance of China (MOF) and MOA issued ‘Provisions on the Administration of the Use of Special Funds for Marine Fishermen Transfer’ in 2003. In 2015, China further adjusted the Fishing Fuel Subsidy Policy, which set a target to reduce fishing fuel subsidy to 40% of the 2014 levels by 2019, in contrast to increasing the ship reduction subsidy from RMB 2500 to RMB 5000 per kilowatts. After that, China’s local governments in succession proposed the specific vessel buyback programs, which put forward a clear objective for the reduction of fishing vessels. Some traditional marine fishermen changed their profession under resource constraints and policy incentives. According to government statistics, the number of traditional fishermen fell by about 7% from 2003 to 2017, and most of them turned to other related industries such as mariculture, fish processing, and recreational fishery [7]. However, in terms of volume, China still has exceeded 3 million traditional marine fishermen. Whether marine fishermen have changed their livelihoods or not, they are facing severe sustainable livelihood challenges generally [8]. On the one hand, because of the high dependence on marine resources and the environment, fishermen who have turned to mariculture, fish processing, recreational fishery, and others are also faced with a livelihood threat from resources recession, marine pollution, and marine disaster [9]. On the other hand, influenced by the characteristics of advanced age and low level of education, fishermen have a strong attachment to the traditional way of life [10]. Therefore, the adaptation to a new profession is challenging for traditional fishermen. Based on this, what is the difference between the livelihood vulnerability of the non-converted fishermen (fishermen who have remained to engage in traditional fishing) and the converted fishermen (fishermen who have converted to other industrial sectors) under the vessel buyback and fishermen transfer programs? What are the differences in livelihood vulnerability among converted fishermen who are engaged in different livelihood strategies? What are the driving factors behind the fishermen’s livelihood vulnerability? Answering these questions is pivotal to making policy decisions on how best to improve the marine fishermen’s livelihood ability and welfare level.

Although some previous studies have attempted to analyze the livelihood challenges faced by Chinese marine fishermen under the vessel buyback and fishermen transfer programs, many are general qualitative studies on a large scale. Studies on the differences in livelihood levels between non-converted fishermen and converted fishermen, as well as within converted fishermen, remain sparse, which is not conducive to the formulation of targeted policies. Thus, a thorough empirical investigation of fishermen’s livelihood and identification of differences in the livelihood levels among fishermen with different means of livelihood are needed.

Internationally, early studies on a marine fishermen’s livelihood have focused on the issue of livelihood sustainability faced by marine fishermen in the context of declining fishery resources due to overfishing [11]. In recent years, numerous studies have attempted to assess the livelihood status of marine fishermen against the impacts of external biophysical drivers (e.g., climate change, storm surges, and ocean acidification) [12,13,14,15]. Besides, a few authors have analyzed the issues of livelihood of marine fishermen in the context of social-ecological dynamics [16]. In general, previous studies could be divided into two different types: vulnerability-based research [17,18,19] and livelihood capital-based research [20,21]. However, studies on Chinese marine fishermen’s livelihood vulnerability and livelihood assets remain sparse or are just a qualitative analysis [22,23].

To address these limitations of extant studies, this paper combined the vulnerability assessment framework and livelihood assets concept into a modified framework of fishermen’s livelihood vulnerability assessment and then conducted an analysis of characteristics and differences underlying the livelihood vulnerability of fishermen with different means of livelihood in the state of Zhoushan, a city of Zhejiang province in China. Finally, the determinant factors of fishermen’s livelihood vulnerability were explored by the regression tree model. The results of this study were useful for policymakers helping fishermen to rebuild a sustainable livelihood.

The remainder of the paper is organized as follows. Section 2 introduces the theoretical framework. Section 3 introduces materials and methods. Section 4 presents the results. Section 5 gives the discussion and implications. We end up with the conclusions in Section 6.

## 2. Theoretical Framework

### 2.1. The Modified Livelihood Vulnerability Framework

According to the definition from the Intergovernmental Panel on Climate Change (IPCC), vulnerability is a function of sensitivity and adaptive capacity of the object of study exposed to a stressor [24]. At present, the framework proposed by the IPCC has been widely adopted for vulnerability assessments, including both individuals’ and communities’ livelihoods. The framework suggests that the vulnerability measure was composed of exposure, sensitivity, and adaptive capacity indicators to identify the relationship between social responses and ecological change [25,26]. Early studies on livelihood vulnerability mainly focused on land farmers. In recent years, the livelihood vulnerability of fishermen under the large-scale environmental changes associated with climate change, marine pollution, and overfishing have attracted wide attention. For example, Jacinto et al. (2014) developed a sector-based fisheries vulnerability assessment tool to evaluate the potential impacts of climate change to commercial fishery sectors or local fishermen in Philippines [17]. Baptiste A K and Kinlocke R (2016) examined the level of vulnerability of different fishers to climate change based on a survey of 241 fishers from Old Harbour Bay [18].

**Figure 1:**
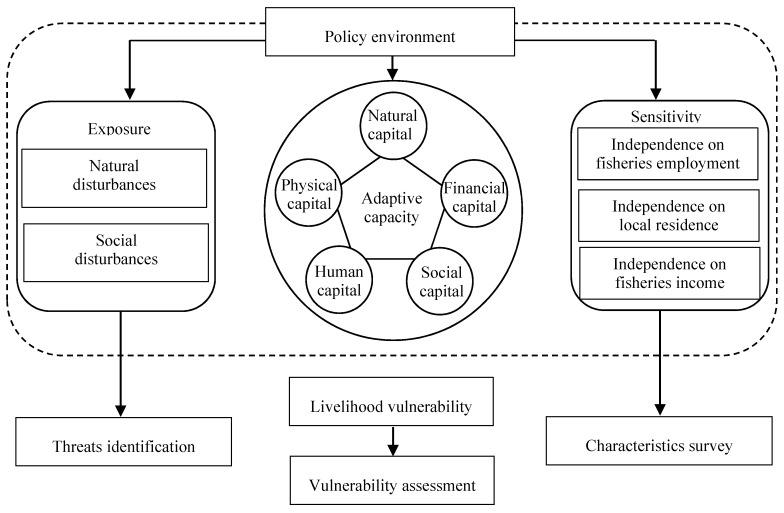
The modified livelihood vulnerability framework focusing on marine fishermen.

The concept of livelihood assets comes from the sustainable livelihood approach, which is a people-centered approach and can be used to set for principles and as an analytical tool to evaluate the level of livelihood of specific groups [27,28,29]. In the light of the sustainable livelihood approach, livelihood is defined as ‘the capabilities, assets, and activities required for means of living’ [30] and livelihood assets comprise five types of capital-natural, physical, financial, human, and social [29,31]. Livelihood assets reflect the capacity of coping and recovering from stress and shocks, which are consistent with the connotation of adaptive capacity in the framework of vulnerability assessment [19,32,33]. Thus, numerous studies adopted or modified the five types of livelihood assets to measure the level of adaptive capacity in the construction of livelihood vulnerability index [34,35,36,37]. Based on existing research, this paper applied the vulnerability assessment framework and sustainable livelihood approach to develop a modified livelihood vulnerability framework for marine fishermen under multiple disturbances (Figure 1). Accordingly, the assessment process of fishermen’s livelihood vulnerability includes external threats identification, fishermen’s characteristics survey, quantitative vulnerability estimation, and sustainable livelihoods construction analysis.

### 2.2. Indicators of Livelihood Vulnerability

Based on the IPCC definition, exposure was defined as the degree of multiple natural and social stresses on marine fishermen’s livelihood [38]. The natural disturbances faced by fishermen mainly included fishery resources recession, marine environment pollution, and marine natural disasters [39,40]. The social disturbances were mainly measured by investigating whether fishermen’s families suffered from property loss, disease, and unemployment [41,42]. Sensitivity indicated the probability of fishermen’s livelihood’s being affected by the impacts of external threats. The sensitivity index was usually measured by employment dependence, residential dependence, and income dependence [43,44]. Exposure and sensitivity represented the potential impacts of a stressor, which were fully experienced in the long-term, depending on the entity’s adaptive capacity [40]. Adaptive capacity was defined as fishermen’s ability to respond to and recover from the potential impact of a stressor. From the perspective of livelihood assets, fishermen’s adaptive capacity could be disaggregated into five categories: natural capital, physical capital, financial capital, human capital, and social capital [45,46].

## 3. Materials and Methods

### 3.1. Study Area

**Figure 2:**
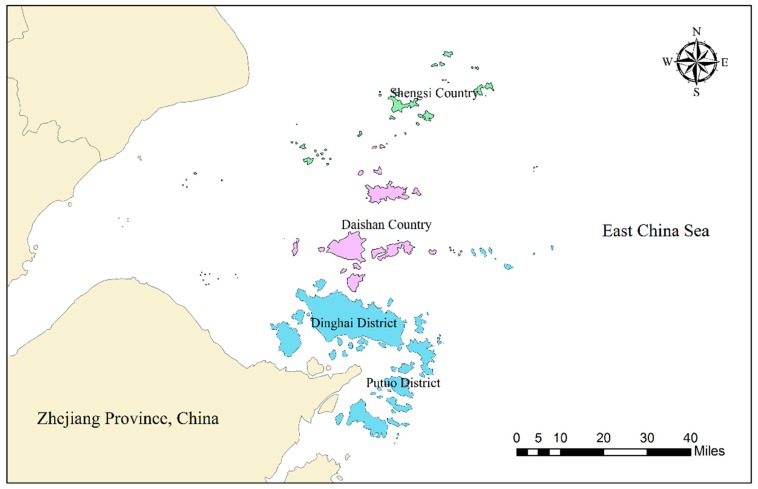
Locations of prefectures considered in this study.

This study examines the livelihood vulnerability of marine fishermen to natural and social disturbances using the Zhoushan, Zhejiang province as a case study, and identifies the differences of livelihood vulnerability among different fishermen under the background of vessel buyback and fishermen transfer programs. Zhoushan is the sole city of an archipelago in China, which has jurisdiction over two districts and two countries: Dinghai District, Putuo District, Daishan Country, and Shengsi Country (Figure 2). Located in the East China Sea, Zhoushan fishing ground is the largest fishing ground in China. Zhoushan’s marine fish production accounted for nearly 10% of China’s total production in recent years [47]. According to government statistics, there were 69,828 fishermen families in Zhoushan, of whom 46,705 fishers were considered as traditional fishermen in 2017, with a decrease of 21,289 compared with 2001 [48]. Most of the traditional fishermen who were no longer engaged in traditional fishing moved to recreational fishery, mariculture, and fish processing. At the same time, a few turned to other industries or chose to retire [49,50]. Therefore, taking Zhoushan as the research area can well meet the needs of this study to explore the livelihood vulnerability differentiation characteristics of different types of fishermen under the vessel buyback and fishermen transfer programs.

### 3.2. Index Formation and Determinants Analysis

Livelihood vulnerability was determined by the combination of exposure, sensitivity, and adaptive capacity. Table 1 shows the components of livelihood vulnerability and the description as well as the weight of each variable. Specifically, the extreme difference method was used to standardize variable values, and the principal components analysis method was adopted for weighting each variable. Then the exposure index, sensitivity index, and adaptive capacity index can be obtained respectively after conducting a weighted summation according to Equation (1):

(1) $$EI,SI,ACI=\sum_{i=1}^{n}W_{i}Y_{i},$$

where *EI*, *SI*, and *ACI* represent the values of exposure index, sensitivity index, and adaptive capacity index, respectively; *W_i_* represents the weight of the *i*th indicator (*i* = 1,2,…, *n*); and *Y_i_* represents the normalized value of the *i*th indicator.

Finally, the fishermen’s livelihood vulnerability index (*LVI*) can be expressed as Equation (2):

(2) LVI=(EI+SI−ACI).

In the calculation of *LVI*, *EI*, *SI*, and *ACI* were treated equally, and each normalized to a 0–1 scale. The K-means cluster analysis method was adopted to divide *LVI* scores into three levels (the high level, medium level, and low level).

Livelihood vulnerability was mainly affected by individual characteristics and livelihood characteristics [21,33,42]. Being based on the method developed by Keshavarz et al. [51], the regression tree model was used for determinants identification in this case study. The dependent variable was LVI, including three levels: high level, medium level, and low level. The independent variables were identified from two aspects: individual characteristics and livelihood characteristics. The former include family size, household income, number of household income sources, education level, and work experiences in the present industry. The latter includes whether they converted or not, the impacts of disturbances, the degree of dependence on fishery income, and whether they were equipped with fishery facilities. Specifically, the impacts of disturbances were measured by the weighted average of marine resource and environment risks as well as household risks. The degree of dependence on fishery income was measured by a ratio of fishery income to total household income. Fishery facilities developed to measure the level of physical capital included fishing vessels, mariculture equipment, and fish processing equipment. The SPSS was used to estimate the regression tree model. The 10-fold cross-validation was adopted to minimize potential biases.

### 3.3. Data Collection

A semi-structured interview and questionnaire were designed to obtain qualitative and quantitative data on individual characteristics (such as age, educational level, and family income) and fishermen’s livelihood vulnerability (including exposure, sensitivity, and adaptive capacity) (see details of the questionnaire in Supplementary Materials). The survey was conducted in Dinghai District, Putuo District, Daishan Country, and Shengsi Country in July and August 2018. Non-converted marine fishermen and converted marine fishermen were the subjects of this survey. Considering that random sampling was difficult to ensure a certain number of converted fishermen were included in survey samples, respondents were identified by a stratified sampling approach in this study. Firstly, with the help of the Administration of Ocean and Fisheries of Zhoushan, 22 administrative villages were selected as sample collection points from four districts and counties, all of which were traditional marine fishing villages of Zhoushan. Then, based on the list of converted fishermen provided by local neighborhood committees and village committees, samples of converted fishermen, and non-converted fishermen were randomly selected according to the proportion of 1:2. Finally, random sampling was conducted again in the sample frame composed of two types of fishermen to identify questionnaire respondents in this study.

### 3.4. Characteristics of Samples

A total of 460 questionnaires were distributed, and 424 valid questionnaires were used for the following analysis, among of which 255 were non-converted fishermen. The characteristics of different types of fishermen were presented in Table 2. Generally, these marine fishermen were older (the average age was over 50 years old) and less educated. Fishermen who were engaged in recreational fishery and traditional marine fishing had relatively higher annual income, while the income of fishermen who have moved to non-fishery industries was lowest. Although part of some fishermen gave up traditional marine fishing as a response to the vessel buyback and fishermen transfer programs, the original level of household income for most of them could not be maintained with an exception for those converting to the recreational fishery. Therefore, it is an urgent issue for the government to help the elderly and low educated fishermen improve their livelihood ability. From the perspective of work experience, the majority of traditional fishermen had more than three years of fishing experience. In contrast, the converted fishermen had much less working experience in the current industry. Therefore, converted fishermen had to face challenges to adapt to the new work in the short term.

## 4. Results

### 4.1. General Analysis of Fishermen’s Livelihood Vulnerability

Based on indicators and methods developed in Section 3, fishermen’s LVI was estimated. F statistics of a one-way analysis of variance was 933.26, and the P statistics was smaller than 0.05, which verified the existence of significant differences among different levels. The proportion of fishermen with a high LVI was about 37.35%, and the average LVI for these fishermen was 0.365. Fishermen with a medium LVI accounted for 41.37% of the total samples, and the average LVI in this group was 0.254. The average LVI for fishermen with a low level of vulnerability was 0.127. As is shown in Table 3, fishermen with high LVI tended to be characterized by advanced age, low level of education, fewer work experiences, lower household annual income, small family size, and a single source of household income. In addition, the converted fishermen contributed to a larger proportion in the group with high LVI than that in groups with medium and low LVI.

### 4.2. Comparison of Livelihood Vulnerability for Different Types of Fishermen

#### 4.2.1. The LVI for Non-Converted and Converted Fishermen

**Figure 3:**
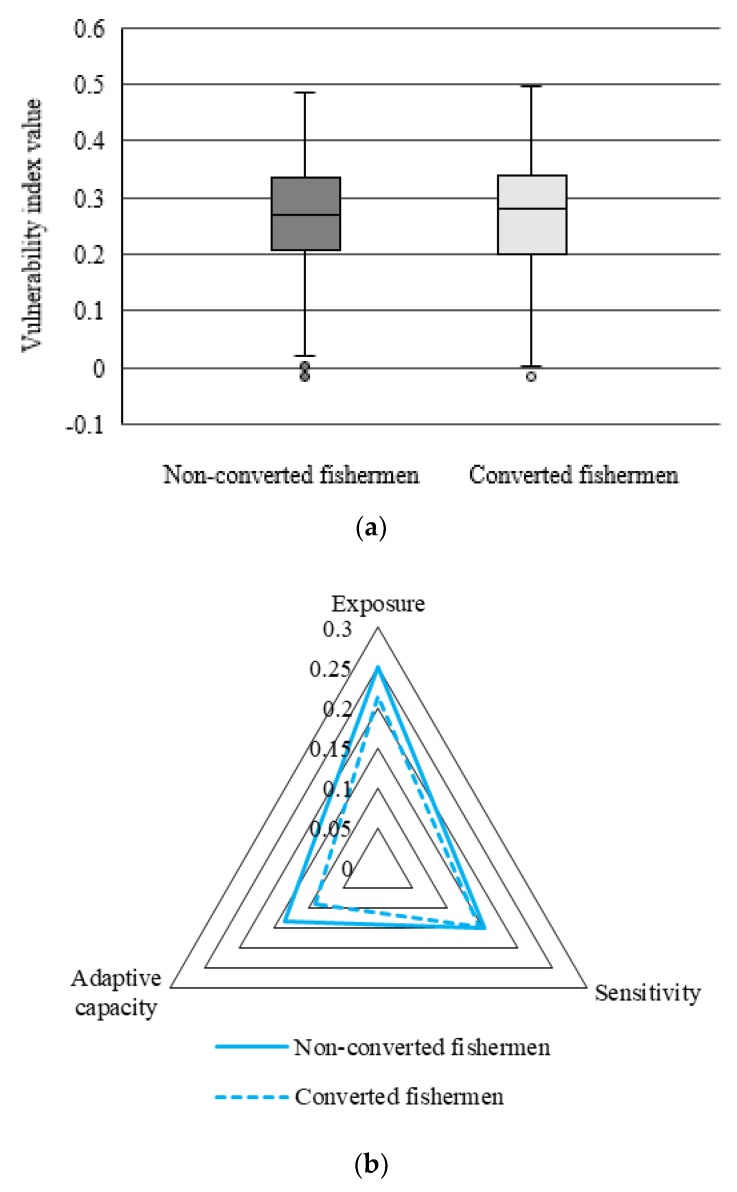
The LVI for non-converted and converted fishermen: (**a**) Box plot of the LVI values; (**b**) Radar chart of the exposure index, sensitivity index, and adaptive capacity index.

The distribution of LVI for both non-converted and converted fishermen was described in Figure 3a. It showed that the interquartile range (IQR) of non-converted fishermen’s LVI distributions was relatively narrow. The median was closed to the middle part of the box body, and the distance between the upper cut-off point and upper quartile was similar to that between the lower cut-off point and the lower quartile. Therefore, the LVI for non-converted fishermen was normally distributed. In addition, there were outliers for non-converted fishermen in the direction of a lower cut-off point, which indicated the livelihood capacity for some of them was significantly higher than that of others. Specifically, according to the survey, fishermen with extremely lower LVI were generally shareholders of fishing vessels. These main shareholders with much higher income are not only endowed with an abundant livelihood capital but also direct beneficiaries of Fishing Fuel Subsidy Policy. Our survey showed that their annual household income was more than 120,000 yuan, far higher than the average household income of all sample fishermen of 833,000 yuan. In contrast, the median of LVI distributions for fishermen who have moved to other industries was more close to the upper quartile, which described a skewed distribution and higher equilibrium tendency. From the perspective of different components of LVI, as shown in Figure 3b, the exposure index, sensitivity index, and adaptive capacity index for converted fishermen were all smaller than that of non-converted fishermen. Specifically, the difference in terms of adaptive capacity was large, while the sensitivity difference was small. The average LVI for converted fishermen was 0.275, which was higher than that of non-converted fishermen, 0.265. Though being suffered from fewer disturbances for fishermen who were engaged in mariculture, fish processing, recreational fishery, or non-fishery industries, the lower adaptive capacity resulted in higher vulnerability for these converted fishermen.

#### 4.2.2. The LVI for Different Types of Converted Fishermen

**Figure 4:**
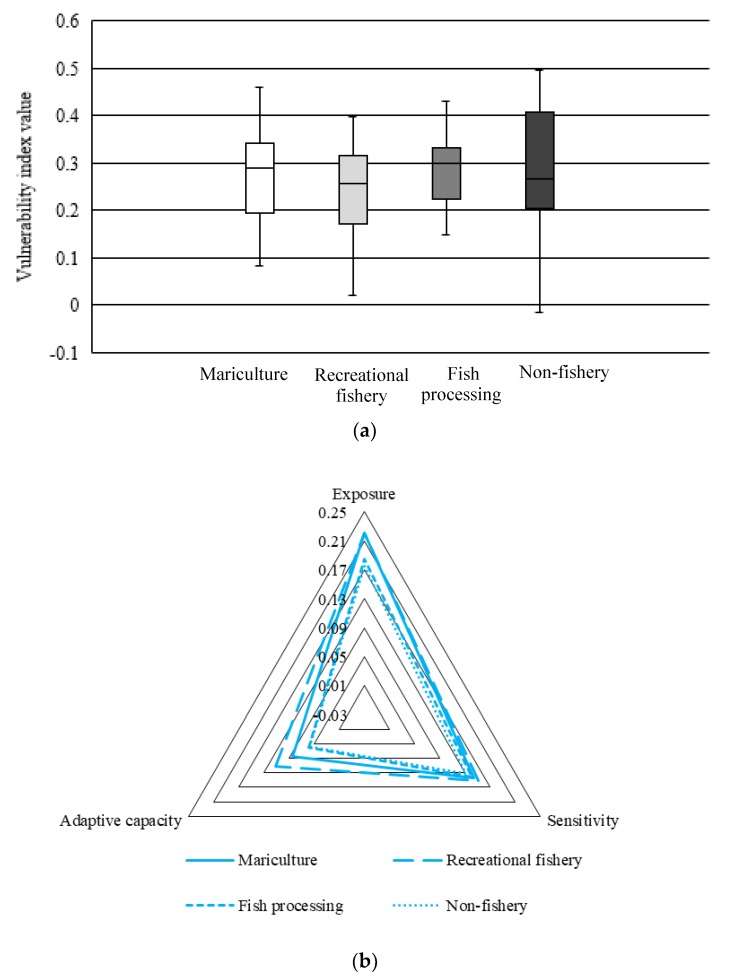
The LVI for different types of converted fishermen: (**a**) Box plot of the LVI values; (**b**) Radar chart of the exposure index, sensitivity index, and adaptive capacity index.

The distribution of LVI for converted fishermen was described in Figure 4a. It showed that the LVI in the mariculture group was between 0.20 and 0.35. The median of LVI in this group was more closed to the upper cut-off point, which indicated a higher equilibrium tendency. Being restricted by technology and facilities, there were only a small proportion of fishermen who converted to mariculture in Zhoushan, and swimming crab, yellow croaker, prawns, and mussels were the main species for cultivation. The general location of LVI distribution for fishermen being engaged in the recreational fishery was lower than that in other groups, which was mainly attributed to a higher adaptive capacity. Furthermore, these families always have higher incomes according to the survey. The distribution of LVI in the fish processing group was narrow, and the median was closer to the upper quartile, indicating that the converted fishermen engaged in the fish processing generally had high livelihood vulnerability. For fishermen who have converted from traditional fishing to the non-fishery industry, the distribution of their LVI was more dispersed than other types of converted fishermen. The survey showed that about 58.13% of converted fishermen who engaged in non-fishery suffered a high level of livelihood vulnerability, and they generally had the characteristics of advanced age, low level of education, and a lack of livelihood capital. In contrast, fishermen with ownership of fishing boats have accumulated an economic basis and social resources in the early stage, so they could better adapt to non-fishery work and had low LVI, but the proportion of these fishermen was only 11.63%. According to our survey, some fishermen with excellent organizational skills and good reputation were elected as village cadre after leaving traditional marine fishing at some sampling points such as Gaoting town in Daishan county and Wulong country in Chengsi county. As a whole, in addition to fishermen who engaged in recreational fishery, the LVI of converted fishermen was generally higher than that of the non-converted fishermen. The main challenge faced by converted fishermen was the advanced age and the lack of livelihood capital.

As shown in Figure 4b, there were significant differences for converted fishermen in terms of exposure degree and adaptive capacity, while the sensitivity index was similar to each other, which indicated that converted fishermen still had a strong attachment to the traditional way of life. Our survey indicated that nearly 85% of the fishermen were reluctant to move out of their places of residence, no matter if they were converted fishermen or non-converted fishermen. At the same time, most of the converted fishermen preferred to engage in other related fishery industries due to the limitation of knowledge and skills. From the perspective of livelihood exposure index, the fishermen engaged in mariculture and recreational fishery had high exposure, while the fishermen engaged in fish processing and non-fishery had low exposure. Similar to traditional fishing, mariculture and recreational fishery were highly dependent on marine environments and resources. Therefore, the converted fishermen who engage in mariculture and recreational fishery were still faced with external risks such as environmental pollution and natural disasters. In contrast, fishermen who have converted to fish processing and non-fishery usually worked as employees with fixed salaries, which resulted in lower exposure. The adaptive capacity of fishermen engaged in the recreational fishery was highest because of the advantages of fishermen in the natural capital, physical capital, and financial capital, which contributed to low LVI. In contrast, the adaptive capacity of fishermen engaged in mariculture was relatively low, which was mainly caused by the low organization level of mariculture in Zhoushan. Most of the fishermen engaged in fish processing and non-fishery had to take temporary jobs in local fish processing companies or became migrant workers after leaving traditional fishing because of poor livelihood capitals. As a result, the adaptive capacity of these two groups was also lower.

### 4.3. The Determinants of Fishermen’s Livelihood Vulnerability

**Figure 5:**
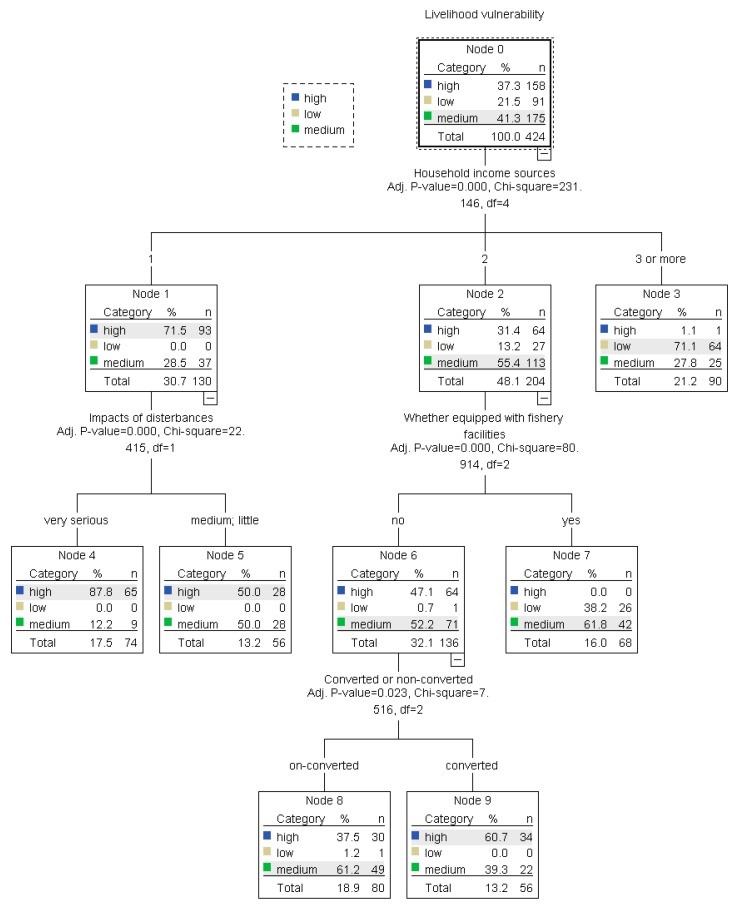
Determinants of marine fishermen’s livelihood vulnerability.

The results of the regression tree model analysis were presented in Figure 5. The number of household income sources, whether they converted or not, impacts of disturbances, and whether they were equipped with fishery facilities, were statistically significant variables. The prediction accuracy for the whole model was 66.5%, and the prediction accuracy for three classified models, the high vulnerability model, the medium vulnerability model, and the low vulnerability model was 80.4%, 52.0%, and 70.3%, respectively. Therefore, the regression tree model could simulate the data set very well. The number of household income sources was the major factor that had an impact on livelihood vulnerability. A lack of alternative income sources was a common challenge faced by fishermen in Zhoushan. It should be noted that the number of household income sources was the single statistically significant factor to determine vulnerability in Node 3, where respondents have more than three sources of income, and 71% of whom were faced with low livelihood vulnerability. It implied that improving the work skills of fishermen and creating alternative income sources were the main solutions to reduce vulnerability.

For respondents in Node 4 and Node 5, the LVI was relatively higher and could be explained mainly by the number of household income sources and impacts of disturbances. Being characterized by single income sources and high dependence on fisheries, these fishermen were easily suffered from marine resource recession, natural disasters, and outside disturbances. For fishermen being faced with extremely high disturbances in Node 4, the proportion with high LVI was about 87.8%. Based on this, it was needed to strengthen vocational training, promote cooperation, and develop a social security system to improve fishermen’s adaptive capacity and rebuild sustainable livelihood. For fishermen in Node 2, whether they have fishery facilities was a key factor in determining the degree of their livelihood vulnerability. It can be known that fishermen being equipped with fishing vessels, mariculture facilities, or fish processing facilities generally had lower livelihood vulnerabilities by a comparison of fishermen in Node 6 and Node 7. Furthermore, for the fishermen in Node 6, whether they chose to change their careers or not was the factor affecting their livelihood vulnerability. The proportion of converted fishermen with high vulnerability in Node 9 was higher than that of non-converted fishermen in Node 8.

## 5. Discussion

### 5.1. Livelihoods Transformation and Livelihood Vulnerability

At present, China’s marine fisheries are facing the challenge of a depletion of fishery resources. In order to restore the fishery resources, the local governments along the coast of China have formulated vessel buyback and fishermen transfer programs to prevent overfishing. However, traditional fishermen generally have the characteristics of advanced age and low level of education, which makes them difficult to adapt to new occupations and face more livelihood threats. Our results showed that the livelihood vulnerability of converted fishermen was generally higher than that of non-converted fishermen, and the low level of adaptive capacity was the main cause of high livelihood vulnerability for converted fishermen. Although the Chinese government has carried out a series of vocational training to ensure that the converted fishermen can adapt to the new occupation as soon as possible, the actual effect of the training was relatively modest because of the low education level and advanced age of the fishermen [52]. Comparing different types of converted fishermen, we found that the livelihood vulnerability of converted fishermen in different livelihood alternatives is also quite different, among which the converted fishermen engaged in the recreational fishery have the lowest level of livelihood vulnerability. Although the converted fishermen engaged in recreational fishery also suffered from exposure risks such as resource recession, environmental pollution, natural disasters, etc., they generally had good livelihood capital and showed higher adaptive capacity. Our survey found that most of these fishermen owned the ownership of fishing boats before they changed careers, so they could get high subsidies from the government after they turned over the fishing boats. The subsidies laid a financial foundation for them to engage in the recreational fishery with high investment and high return. In contrast, the livelihood vulnerability level of the converted fishermen who were engaged in fish processing and non-fishery work was relatively high. These converted fishermen did not get the ship reduction subsidies but also lacked physical capital for aquaculture and recreational fishery, and they could only choose to work in the local aquatic products processing enterprises or other enterprises to get a lower wage income. Therefore, in the process of livelihood transformation, the marine fishermen’s livelihood vulnerability showed an inertial characteristic, that is, the adaptive capacity of fishermen who lacked livelihood capital has not changed after their transition, and their livelihood vulnerability level was still high. In contrast, the adaptive capacity of fishermen who were rich in livelihood capital was still high, and their livelihood vulnerability level remained low.

### 5.2. Implications for Improving Fishermen’s Livelihood

Several suggestions can be proposed to build a sustainable livelihood for marine fishermen based on the above analysis. First, develop a vessel share cooperation system. The owners of fishing vessels should be encouraged to sign a cooperation contract with their employees. On the one hand, the management right, as well as the right to earnings of the common fishermen can be guaranteed. Accordingly, these fishermen can be endowed with a higher level of livelihood capacity. On the other hand, risks faced by owners caused by marine resource recession, environment pollution as well as other disasters can also be shared with common fishermen. Second, improve the technical training system. Develop different types of production training courses based on fishermen’s livelihood capital as well as local resource endowment, which aims to improve fishermen’s adaptive capacity. Third, encourage vessel owners with a high reputation to join the village committee or fishery cooperation organizations with the expectation to help local fishermen transfer to mariculture, recreational fishery, or other sustainable production activities. Fourth, improve the endowment insurance system and encourage fishermen to participate. In addition, special subsidy policies should be developed for fishermen in advanced age or disability.

### 5.3. Comparisons with Other Studies

Many studies have proved that fishermen’s livelihood was affected by multiple natural disturbances such as climate change, resource decline, environmental pollution, and presented high vulnerability [16]. This study considered these natural indicators in addition to social disturbances when considering exposure factors, and developed a framework of fishermen’s livelihood vulnerability assessment under social-ecological stresses. The empirical results of this paper also confirmed that marine fishermen were generally faced with greater livelihood pressure, and more than one-third of fishermen had a high level of livelihood vulnerability. In terms of sensitivity, the family income of fishermen was generally highly dependent on the fishery, and the source of fishermen’s income was relatively single, which greatly increased the livelihood vulnerability of fishermen. Similar to our results, studies in marine protected areas also found that the fishermen’s livelihood vulnerability was affected by dependency on the fishery and availability of alternative fisheries [45]. In addition, the unpredictability of fishery income might also increase the fishermen’s sensitivity [21].

Fishermen’s livelihood capital was considered to be the key factor in effectively improving the ability of risk prevention, which was another focus of the current research. In various situations, different types of livelihood capital had different effects on improving adaptive capacity. For example, research on the livelihood of fishing areas under the disturbance of climate change showed that fishermen with a high level of social capital were more sensitive to external risk, and they could obtain risk information faster and adjust their livelihood strategies in time [14]. Research in fishing communities in transition indicated that a lack of financial capital was an important reason for the livelihood crisis faced by fishermen in the process of transition, and most fishing households have taken loans several times higher than what they earn [5]. Our study compared the adaptive capacity of different types of fishermen, in which the inadequate physical capital and financial capital were the main reason for the high level of livelihood vulnerability.

### 5.4. Prospects for Research

Our study focused on the analysis of characteristics and differences underlying the livelihood vulnerability of fishermen with different means of livelihood but did not reveal the mechanism of vulnerability. The livelihood strategies adopted by fishermen were related to the exposure, sensitivity, and adaptive capacity, especially influenced by the livelihood capital [20], and the choice of different livelihood strategies would lead to different results of livelihood vulnerability [22]. Therefore, the identification of the interaction between livelihood capital, livelihood strategies, and livelihood vulnerability should be a focus of future research.

## 6. Conclusions

Being based on vulnerability theory and the concept of livelihood assets in the framework of sustainable livelihood, a framework to assess the livelihood vulnerability of marine fishermen was developed from exposure, sensitivity, and the adaptive capacity three aspects. In this study, 424 fishermen, including converted ones and non-converted ones, as well as different types of converted fishermen, were interviewed to conduct the empirical analysis. Moreover, the regression tree model was developed to identify determinants of fishermen’s livelihood vulnerability. Our study revealed that fishermen with a high level of vulnerability accounted for about one-third, and they were characterized by advanced age, low level of education, a lack of work experience, low family income, fewer family members, and fewer income sources. The livelihood vulnerability for converted fishermen was higher than that for non-converted fishermen, mainly because of relatively lower adaptive capacity. There were also differences in livelihood vulnerability for different types of converted fishermen. Specifically, the fishermen who engaged in the recreational fishery had the lowest vulnerability. The analysis of the regression tree model indicated that fishermen’s livelihood vulnerability was mainly influenced by many factors, such as fishery facilities, number of household income sources, and impacts of disturbances. In the process of sustainable livelihood reconstruction, the government should pay more attention to the converted fishermen who have a single income source and a lack of physical capital and financial capital.

## Figures and Tables

**Table 1:** Livelihood vulnerability dimension, component, and index description, and code, mean, and standard deviation of indicators.

Dimension	Component	Index Measure	Weights	Code	Mean	Standard Deviation
Exposure	Natural disturbances	Impacts from fishery resources recession	0.064	Very serious = 5, serious = 4, modest = 3, little = 2, no impacts = 1	3.897	1.331
		Impacts from marine environment pollution	0.060	Very serious = 5, serious = 4, modest = 3, little = 2, no impacts = 1	3.422	1.180
		Impacts from marine natural disasters	0.058	Very serious = 5, serious = 4, modest = 3, little = 2, no impacts = 1	3.572	1.101
	Social disturbances	Loss of property	0.054	Yes = 1, No = 0	0.348	0.467
		Suffer from a major illness	0.052	Yes = 1, No = 0	0.125	0.304
		Lost jobs	0.050	Yes = 1, No = 0	0.189	0.312
Sensitivity	Dependence on fisheries employment	Proportion of family labors who are engaged in fishery industries	0.060	Number of family labors who are engaged in fishery industries/Total family labors	0.692	0.764
	Dependence on local residence	Whether want to leave hometown if you have the chance	0.059	Yes = 0, No = 1	0.263	0.342
	Dependence on fisheries income	Proportion of fishery income to total family income	0.058	Very high = 4, high = 3, small = 2, very small = 1	3.303	0.799
Adaptive Capacity	Natural capital	Marine space size for fishery production	0.048	Very large = 5, large = 4, modest = 3, small = 2, very small = 1	3.353	2.243
	Physical capital	The number of housing rooms	0.059	One room = 1, two rooms = 2, three rooms = 3, four or more rooms = 4	2.994	1.269
		Whether there are fishing vessels	0.043	Yes = 1, No = 0	1.434	0.845
		Whether there are mariculture or fish processing equipment	0.042	Yes = 1, No = 0	1.994	1.464
	Financial capital	The opportunities of getting fishery subsidies from government	0.040	A great many = 4, many = 3, few = 2, very few = 1	2.450	0.965
		The degree of difficulties of getting loans from markets	0.039	Very easy = 4, easy = 3, difficult = 2, very difficult = 1	2.109	1.285
		The degree of difficulties of getting financial support from relatives and friends	0.033	Very easy = 4, easy = 3, difficult = 2, very difficult = 1	2.983	1.904
	Human capital	The number of youth labor force (age between 20–59) in family	0.043	Natural number	1.456	0.707
		Whether have obtained some vocational and technical training	0.039	Yes = 1, No = 0	0.306	0.501
	Social capital	Whether there are family members serving as village cadre	0.037	Yes = 1, No = 0	0.117	0.355
		Whether is member of fishery co-operative or other fishery associations	0.033	Yes = 1, No = 0	0.225	0.418
		Number of relatives or friends who can help you in daily life	0.029	Natural number	2.358	1.893

**Table 2:** Characteristics of converted and non-converted fishermen.

Characteristics	Non-Converted	Converted			
	Marine Fishing	Mariculture	Recreational Fishery	Fish Processing	Non-Fishery
Family size (person)	4.05	4.22	4.17	3.65	4.78
Age (year)	55.99	53.29	52.45	54.5	52.87
Household income (10,000 RMB)	8.56	7.75	9.97	7.25	6.8
Level of education (%)					
Illiterate	8.8	7.69	5.26	6.67	13.95
Primary school	47.54	38.46	38.6	63.33	44.19
Junior middle school	34.15	43.59	49.12	23.33	30.23
Senior middle school or above	9.51	10.26	7.02	6.67	11.63
Work experiences in present industry (%)					
Less than one year	0	10.25	15.79	20	37.21
One to three years	5.89	38.46	38.6	46.67	39.53
More than three years	94.11	51.29	45.61	33.33	23.26

**Table 3:** Characteristics of fishermen with different levels of the livelihood vulnerability index (LVI; mean values).

Characteristics	Description	Total Sample	High Level	Medium Level	Low Level
Age	Year	55.05	55.81	55.03	53.73
Family size	Number of family members	4.14	4.01	4.19	4.27
Education level	Illiterate = 0, Primary school = 1, Junior middle school = 2, Senior middle school or above = 3	1.45	1.26	1.54	1.61
Work experiences in present industry	Less than one year = 0, One to three years = 1, More than three years = 2	1.62	1.49	1.65	1.77
Household income	Ten thousand Yuan per year	8.33	7.81	8.48	8.96
Number of household income sources	Natural number	2.01	1.38	2.28	2.54
Converted or not	Non-converted = 1, converted = 0	0.67	0.59	0.68	0.79

## Supplementary Materials

The following are available online at https://www.mdpi.com/1660-4601/17/3/765/s1.
