# Supplementary material for: Livelihood Vulnerability of Marine Fishermen to Multi-Stresses under the Vessel Buyback and Fishermen Transfer Programs in China: The Case of Zhoushan City, Zhejiang Province
Source: Int J Environ Res Public Health. 2020 Jan 25;17(3):765. doi: 10.3390/ijerph17030765 (PMC7037923; doi:10.3390/ijerph17030765)
Supplement: Supplementary file 1 [file ijerph-17-00765-s001.pdf]

Supplementary

A survey on the marine fishermen’s livelihood vulnerability in Zhoushan City, Zhejiang Province

Dear respondents,

We are a member of the Fishermen's Livelihood Research group of Ningbo University. As a part of our Marine Fishermen’s Livelihood Vulnerability Research assignment, we are hereby conducting a questionnaire to investigate fishermen’s livelihood strategies, livelihood ability and welfare level. We could highly appreciate if you can take a few minutes to complete this questionnaire. Information received will be used for academic research purposes. All of your answers will be kept strictly confidential.

Thank you for participating in this questionnaire.

Section 1—Basic information

1. Your gender：

A. Male B. Female

2. Your Age: __________.

3. How many people are there in your family? __________.

4. How much is your annual household income? __________(unit: 10000 RMB).

5. What's your education level?

A. Illiterate B. Primary school C. Junior middle school D. Senior middle school or above

6. What is your current job?

A. Traditional fishing B. Mariculture C. Recreation fishery D. Fishing processing E. Non-fishery

7. How many years have you done in your current job?

A. Less than one year B. One to three years C. More than three years

Section 2—Livelihood vulnerability

Part A: Exposure

1. What is the impact of the decline of fishery resources on your family's production and life?

A. Very serious B. Serious C. Modest D. Little E. No impacts

2. What is the impact of the marine environment pollution on your family's production and life?

A. Very serious B. Serious C. Modest D. Little E. No impacts

3. What is the impact of the marine natural disasters on your family's production and life?

A. Very serious B. Serious C. Modest D. Little E. No impacts

4. Has your family suffered property damage in the past year?

A. Yes B. No

5. Have you or any of your family members had a major illness in the past year?

A. Yes B. No

6. Have you or any of your family members lost their jobs in the past year?

A. Yes B. No

Part B: Sensitivity

1. Number of labors in your family: __________. Number of labors who are engaged in fishery industries in your family: __________.

2. If there is an opportunity, would you like to move out of your hometown?

A. Yes B. No

3. What is the proportion of fishery income to total household income?

A. Very high B. High C. Small D. Very small

Part C: Adaptive capacity

1. Do you think there is enough space for you to engage in fishery production?

A. Very large B. large C. modest D. small E. very small

2. How many rooms do you have for your family?

A. One room B. Two rooms C. Three rooms D. Four or more rooms

3. Does your family own a fishing boat?

A. Yes B. No

4. Does your family have equipment for aquaculture or aquatic products processing?

A. Yes B. No

5. What are your chances of getting fishery subsidies from the government?

A. A great many B. Many C. Few D. Very few

6. How difficult is it for you to get a loan from the market?

A. Very easy B. Easy C. Difficult D. Very difficult

7. How difficult is it for you to get financial support from relatives and friends?

A. Very easy B. Easy C. Difficult D. Very difficult

8. Number of youth labor force (age between 20-59) in your family: _________.

9. Have you obtained some vocational and technical training?

A. Yes B. No

10. Do you or any of your family members serve as village cadres?

A. Yes B. No

11. Are you a member of a fishery cooperative or other fishery association?

A. Yes B. No

12. Number of relatives and friends who can help you in daily life: _________.

Thank you for your kind cooperation !
